# Death and Dying Attitude Through the Eyes of Nursing Students during Clinical Training: A Cross-Sectional Study in the United Arab Emirates

**DOI:** 10.1177/26892820251395427

**Published:** 2025-11-17

**Authors:** Amina Elzeiny, Ahmed Loutfy, Corrien Van Belkum, Hussein M. Magdi, Alaa Elbatanouny, Bushra Al Hariri, Haneen Alazazmeh, Abrar Alrefai

**Affiliations:** ^1^ Department of Nursing, College of Dentistry and Health Sciences, University of Fujairah, UAE.; ^2^ Department of Pediatric Nursing, Faculty of Nursing, Beni-Suef University, Egypt.; ^3^ North London NHS Foundation Trust, London, England.

**Keywords:** clinical training, death, dying, emotional impact, end-of-life care, nursing students, psychological impact

## Abstract

**Background:**

Nursing students often encounter emotional challenges when facing death during their clinical training, which can negatively impact their academic performance and mental well-being. As they provide crucial support to patients and their families, this responsibility can induce significant stress.

**Objective:**

This study aims to examine nursing students’ attitudes toward death and dying and to identify the demographic and educational factors influencing their preparedness for end-of-life care in the United Arab Emirates context.

**Methods:**

In March 2025, a cross-sectional study was conducted using a convenience sample of 122 nursing students (senior and junior) enrolled in clinical placements at a university in the United Arab Emirates. Data collection utilized the validated Death Attitude Profile-Revised (DAP-R) and Frommelt Attitudes Toward Care of the Dying (FATCOD) scales as assessment tools.

**Results:**

The study showed that nursing students typically hold neutral attitudes toward caring for patients nearing the end of life. There are statistically significant correlations between age, education level, grade point average, clinical scores, and overall scores on the DAP-R scale, as well as the FATCOD Scale, among the participants.

**Conclusions:**

While many students showed some acceptance of death, high degrees of fear, avoidance, and escape acceptance had a significant impact on their views about end-of-life care. The findings indicate that emotional distress and insufficient training may act as impediments to compassionate treatment. Furthermore, the comparatively low FATCOD ratings indicate a need for better integration of palliative care instruction within the nursing curriculum. Structured clinical exposure, psychological coping mechanisms, and reflective learning opportunities could all be used to help students gain the essential confidence and emotional resilience.

## Key Message

This study highlights the neutral attitudes of nursing students toward end-of-life care and the emotional challenges they face. It underscores the need for improved palliative care training and support systems to foster compassionate treatment and enhance both students’ mental well-being and their ability to care for dying patients.

## Introduction

Nursing students often embark on clinical training as a crucial part of their education, allowing them to bridge the gap between theoretical knowledge and practical application. However, this phase of their learning journey is not without challenges. One of the most profound and emotionally taxing experiences they encounter is witnessing dying and death. For many, these moments are their first direct exposure to the realities of mortality, which can lead to significant emotional and psychological stress. The weight of caring for patients in their final moments, coupled with feelings of helplessness or inadequacy, can deeply impact their mental well-being and shape their perspective on the nursing profession. Recognizing and addressing these stresses is essential to ensure their growth and resilience as future health care providers.^1^

Among the many difficulties nursing students encounter in their clinical rotations is the possibility of confronting death and dying. Nursing students face significant emotional and psychological challenges as they participate in real-world clinical scenarios, frequently interacting with patients who are nearing the end of their lives. These experiences have been linked to elevated anxiety, mental discomfort, and a sense of powerlessness, according to studies.^2^ These emotional strains can impact students’ academic and professional development and mental health. Anxiety and emotions of vulnerability have been observed to worsen when clinical practice is conducted without enough planning and support.^3^

Exposure to death and dying also highlights the difficulties nursing students face on a personal and professional level. Nursing students may suffer from compassion fatigue, emotional tiredness, and even symptoms similar to post-traumatic stress disorder as they manage the difficult emotional reactions to patients who are near death.^4^ On the contrary, when supported by organized support networks, these experiences can also facilitate personal development. Students can have a deeper understanding of palliative care, enhanced communication skills, and increased emotional resilience with the help of appropriate debriefing, mentorship, and reflective practice. According to research, nursing students can enhance their professional identity and sympathetic ability by favorably adjusting to the problems of death and dying if they receive the necessary assistance.^5^

Nursing students are affected emotionally and psychologically by their experiences with death and dying during their training but despite the importance of being aware of that, only a limited amount of research has explored this area. For instance, one study conducted by Szczupakowska et al.^6^ recognized that 81.9% of the nursing students involved felt unprepared for death and dying circumstances. Similarly, Edo-Gual et al.^7^ mentioned that 75% of the students felt emotionally unprepared to handle such cases since most of the responses indicated a feeling of stress and anxiety. The review indicates that there are simply not enough extended studies to inform solutions, as evidenced by another study conducted by Sampaio et al.^8^ reported the level of emotional distress that nursing students experienced following exposure to a patient’s death in clinical settings, due to the lack of support and education around the topic. Although the experience appears to be universal across cultures,^7,9^ the strategies that nursing students use to overcome the psychological demands of death and dying have received little attention. Students often cope in isolation as very few courses are designed to offer specialized services such as grief counseling or training on emotional preparedness.^10^ This identifies a need for further exploration to bridge the gap between emotional preparedness and technical competence and the development of educational programs.

From a global perspective, the landscape of nursing education—particularly in palliative care—reveals significant variations in both duration and content. In the United Arab Emirates (UAE), as in many countries across the Middle East, the integration of palliative care within undergraduate nursing curricula remains limited. Most nursing programs offer only minimal theoretical exposure, often without structured clinical placements in palliative care units. Consequently, many students’ first encounters with death and dying occur incidentally during general clinical rotations, rather than as part of a guided learning process.^10^

Alarmingly, many newly graduated nurses find themselves inadequately prepared to face death and provide compassionate care for patients at the end of life. Given that nearly all nurses will encounter patients in these critical moments, it’s often the first exposure to death for many nursing students. Therefore, addressing this gap in education is crucial. Comprehensive training in end-of-life care is essential not only for the professional development of nurses but also for ensuring that patients receive the compassionate support they deserve during their most vulnerable times.^11^ This study seeks to investigate the nursing students’ attitudes toward death and dying in the clinical setting.

## Methods

### Design

This study employs a cross-sectional observational design to assess attitudes toward death and dying among undergraduate junior and senior nursing students enrolled in clinical courses at a university in the UAE. Adherence to the Strengthening the Reporting of Observational Studies in Epidemiology checklist for cross-sectional studies was consistently maintained throughout the research.

### Sample

The sample size is determined based on the total target population (156 students) of eligible participants within the nursing program. Using the Krejcie and Morgan sample size determination method, a minimum sample size of 113 students is projected, based on an expected response rate of 50%, a 10% margin of error, and a 99% confidence level.^12^ The response rate for the survey was approximately 78.2%, with 122 out of 156 students completing the survey.

### Data collection tools

The current study utilized two validated survey instruments for data collection: the Frommelt Attitudes Toward Care of the Dying (FATCOD) Scale^13^ and the Death Attitude Profile-Revised (DAP-R).^14^ These instruments, along with a demographic questionnaire, were distributed via the Google Forms platform to all 156 students through their official school e-mail during the spring semester of 2025 (March 2025). Data were collected anonymously and used to explore potential associations between demographic variables and study outcomes. The questionnaire was pilot tested for clarity and relevance prior to administration.

Tool 1: A structured sociodemographic questionnaire was developed to capture key participant characteristics relevant to the study context. The tool included items on age, gender, nationality, marital status, academic year, and GPA score.

Tool 2: The DAP-R is designed to evaluate students’ attitudes toward death and encompasses five subdomains: (a) fear of death, (b) death avoidance, (c) neutral acceptance, (d) approach acceptance, and (e) escape acceptance. Comprising 32 items, responses are scored on a 7-point Likert scale ranging from “Strongly Disagree” to “Strongly Agree,” with average scores calculated for each dimension. The DAP-R has demonstrated high reliability, with Cronbach’s α for the subdomains ranging from 0.61 to 0.97.^14^ In this study, internal consistency was found to be between 0.617 (neutral acceptance) and 0.917 (escape acceptance). The total Cronbach’s α was 0.941.

Tool 3: The FATCOD Scale measures attitudes toward providing care for patients nearing the end of life and is regarded as a reliable tool. For this study, FATCOD Form B will be employed, consisting of 30 items rated on a 5-point Likert scale (Strongly Disagree, Disagree, Uncertain, Agree, and Strongly Agree). The scale includes 15 positively worded statements and 15 negatively worded statements, with the latter inverted during analysis. Higher scores reflect more positive attitudes toward end-of-life care, with total scores ranging from 30 to 150. The FATCOD Scale has demonstrated good reliability, evidenced by a Cronbach’s α of 0.81,^13^ and this study reported an internal consistency of 0.875.

### Data analysis

Data were collected and analyzed using the Computer Statistical Package for Social Science SPSS software (version 25), employing descriptive statistics in the form of frequencies and percentages, and inferential tests including Pearson correlation and independent-samples *t* test. A multiple linear regression analysis was performed to identify predictors of students’ willingness to care for dying patients, as measured by FATCOD scores. Independent variables included the five subscales of the DAP-R. Statistical significance was set at *p* ≤ 0.05.

### Ethical consideration

The scientific research ethics committee of the University of Fujairah, UAE, approved the study (Approval No. CHS-CRP-SA–IW#5/EA#9). Before obtaining written consent from participants via Google Forms, all study information and the roles of the students were clearly outlined. Participants were informed about the study’s purpose, procedures, withdrawal instructions, and confidentiality. All ethical standards for scientific research were adhered to in this study.

## Results

The study sample is predominantly comprised of young adults (73%) and females (68.9%) (Table 1), which aligns with typical nursing student demographics. The high percentage of participants had an “Excellent” grade point average (GPA) (57.4%), and most were single (93.4%).

**Table 1.** Sociodemographic Characteristics Among Studied Subjects (*N* = 122)

Item	No.	Percentage
Age		
Teenagers (≤19 years)	3	2.5
Young adults (20–24 years)	89	73.0
Adults (25–29 years)	28	23.0
Older adults (30+ years)	2	1.6
Mean ± SD	25.39 ± 3.32	
Gender		
Male	38	31.1
Female	84	68.9
Nationality		
Syrian	32	26.2
Egyptian	19	15.6
Emirati	18	14.8
Jordanian	12	9.8
Other	41	33.5
Marital status		
Single	114	93.4
Married	7	5.7
Divorce	1	0.8
BSN level		
BSN 3	64	52.5
BSN 4	58	47.5
GPA score		
Excellent (3.7–4.0)	70	57.4
Very good (3.0–3.69)	38	31.1
Good (2.0–2.99)	10	8.2
Mean ± SD	3.61 ± 0.45	
Did you face death or dying in your clinical training?		
No	36	29.5
Yes	86	70.5

BSN, bachelor of science in nursing; GPA, grade point average; SD, standard deviation.

In regard to DAP-R, most participants score high on neutral and approach acceptance and moderate on fear of death and death avoidance (Table 2), reflecting an overall positive attitude but with some remaining discomfort regarding death. Meanwhile, a low percentage (26.2%) of students scored ≥60% on FATCOD (Table 3), which suggests that while many students accept death, fewer feel fully prepared to care for dying patients.

**Table 2.** Total Score of Death Attitude Profile-Revised Scale Among Studied Subjects (*N* = 122)

Item	Mean	SD	Range	<60%	≥60%
Fear of death	35.84	8.99	14–49	39 (32.0)	83 (68.0)
Death avoidance	26.05	6.88	7–35	35 (28.7)	87 (71.3)
Neutral acceptance	29.41	4.04	16–35	9 (7.4)	113 (92.6)
Approach acceptance	56.24	11.03	21–70	20 (16.4)	102 (83.6)
Escape acceptance	24.46	8.04	5–35	46 (37.7)	76 (62.3)
Total	172.00	30.51	107–224	29 (23.8)	93 (76.2)

**Table 3.** Total Score of Frommelt Attitude Toward Care of the Dying Scale Among Studied Subjects (*N* = 122)

Item	Mean	SD	Range	<60%	≥60%
Total FATCOD	96.81	11.90	72–143	90 (73.8)	32 (26.2)

FATCOD, Frommelt Attitudes Toward Care of the Dying.

Fear of death (*r* = −0.384, *p* < 0.001) and death avoidance (*r* = −0.506, *p* < 0.001) negatively correlate with FATCOD scores (Table 4), suggesting that students with higher fear or avoidance of death have more negative attitudes toward caring for dying patients. Escape acceptance (*r* = −0.322, *p* < 0.001) also shows a negative correlation, indicating that students who view death as an escape struggle more with end-of-life care. In addition, neutral acceptance (*r* = 0.130, *p* = 0.153) and approach acceptance (*r* = −0.118, *p* = 0.197) are not significantly correlated, suggesting that a neutral view of death does not strongly predict attitudes toward patient care programs to address attitudes toward death to improve the care for dying patients among health care professionals.

**Table 4.** Correlation Between Total Score of Death Attitude Profile-Revised Scale and Frommelt Attitude Toward Care of the Dying Scale Among Studied Subjects (*N* = 122)

DAP-R	FATCOD	
	*r*	Sig.
Fear of death	−0.384	0.000^*^
Death avoidance	−0.506	0.000^*^
Neutral acceptance	0.130	0.153
Approach acceptance	−0.118	0.197
Escape acceptance	−0.322	0.000^*^
DAP-R total	−0.337	0.000^*^

^*^ Statistically significant at *p* ≤ 0.01.

DAP-R, Death Attitude Profile-Revised; *r*, Pearson correlation; Sig., Significance.

Furthermore, higher scores of fear of death, death avoidance, and escape acceptance are associated with lower FATCOD scores (*p* < 0.05) (Table 5). Also, neutral acceptance and approach acceptance do not show significant differences, indicating that accepting death does not necessarily translate into higher competence in end-of-life care.

**Table 5.** Relation Between Total Score of Death Attitude Profile-Revised Scale and Frommelt Attitude Toward Care of the Dying Scale Among Studied Subjects (*N* = 122)

DAP-R	FATCOD		
	No. (Percentage)	Mean ± SD	*t* (Sig.)
Fear of death	<60%—90 (73.8)	30.34 ± 9.41	−4.32 (0.000^**^)
	≥60%—32 (26.2)	37.80 ± 8.01	
Death avoidance	<60%—90 (73.8)	21.06 ± 7.79	−5.28 (0.000^**^)
	≥60%—32 (26.2)	27.82 ± 5.57	
Neutral acceptance	<60%—90 (73.8)	30.03 ± 3.12	1.01 (0.313)
	≥60%—32 (26.2)	29.19 ± 4.32	
Approach acceptance	<60%—90 (73.8)	54.97 ± 9.83	−0.76 (0.451)
	≥60%—32 (26.2)	56.69 ± 11.45	
Escape acceptance	<60%—90 (73.8)	20.84 ± 9.04	−3.06 (0.003^**^)
	≥60%—32 (26.2)	25.74 ± 7.28	
DAP-R total	<60%—90 (73.8)	157.25 ± 20.99	−3.31 (0.001^**^)
	≥60%—32 (26.2)	177.24 ± 31.72	

*t*, independent-samples *t* test.

^*^ Statistically significant at *p* ≤ 0.05.

^**^ Statistically significant at *p* ≤ 0.01.

In addition, females score significantly higher on death avoidance (*p* = 0.043) (Table 6), suggesting they tend to avoid thinking about death more than males. However, no significant gender differences appear in fear of death, neutral acceptance, approach acceptance, or escape acceptance, indicating that both genders have similar overall attitudes toward death and end-of-life care. Moreover, there is a statistically significant correlation between age, level, GPA, clinical score, and DAP-R and FATCOD scales’ total scores among the studied subjects.

**Table 6.** Gender Difference in Attitudes as Reported in Death Attitude Profile-Revised Scale and Frommelt Attitude Toward Care of the Dying Scale Among Studied Subjects (*N* = 122)

Item	Gender	*N* (%)	Mean ± SD	*t* (sig.)
Fear of death	Male	38 (31.1)	34.32 ± 9.81	−1.27 (0.208)
	Female	84 (68.9)	36.54 ± 8.56	
Death avoidance	Male	38 (31.1)	24.18 ± 7.67	−2.04 (0.043^*^)
	Female	84 (68.9)	26.89 ± 6.36	
Neutral acceptance	Male	38 (31.1)	29.61 ± 4.20	0.36 (0.721)
	Female	84 (68.9)	29.32 ± 3.99	
Approach acceptance	Male	38 (31.1)	56.95 ± 10.63	0.48 (0.635)
	Female	84 (68.9)	55.92 ± 11.26	
Escape acceptance	Male	38 (31.1)	25.63 ± 6.83	1.09 (0.280)
	Female	84 (68.9)	23.93 ± 8.51	
DAP-R total	Male	38 (31.1)	170.68 ± 30.53	−0.32 (0.750)
	Female	84 (68.9)	172.60 ± 30.67	
FATCOD total	Male	38 (31.1)	96.50 ± 12.64	−0.19 (0.847)
	Female	84 (68.9)	96.95 ± 11.62	

^*^ Statistically significant at *p* ≤ 0.05.

While age did not significantly predict FATCOD scores, it did have a strong correlation with a number of DAP-R subscales (Table 7). This implies that while older students may have a different perspective on death—exhibiting greater degrees of avoidance, approach acceptance, and escape acceptance—their willingness or perceived ability to provide end-of-life care may not be impacted by these variations. One plausible reason is that the nursing program’s collective cultural and educational experiences reduce the impact of age on attitudes toward actual caregiving.

**Table 7.** Correlation Between Age, Level, GPA, Clinical Score, and Total Score of Death Attitude Profile-Revised Scale and Frommelt Attitude Toward Care of the Dying Scale Among Studied Subjects (*N* = 122)

	Age		BSN level		GBA	
	*r*	Sig.	*r*	Sig.	*r*	Sig.
Fear of death	0.287	0.001^**^	0.423	0.000^**^	0.140	0.123
Death avoidance	0.231	0.010^*^	0.427	0.000^**^	−0.007	0.935
Neutral acceptance	0.062	0.497	0.150	0.100	0.072	0.431
Approach acceptance	0.350	0.000^**^	0.566	0.000^**^	0.217	0.016^*^
Escape acceptance	0.381	0.000^**^	0.532	0.000^**^	0.188	0.038^*^
DAP-R total	0.372	0.000^**^	0.585	0.000^**^	0.177	0.051
FATCOD total	−0.170	0.061	−0.306	0.001^**^	−0.036	0.696

^*^ Statistically significant at *p* ≤ 0.05.

^**^ Statistically significant at *p* ≤ 0.01.

With respect to the regression analysis (Table 8), it indicated that death avoidance (*p* < 0.001) and escape acceptance (*p* = 0.016) were significant negative predictors of attitudes toward caring for dying patients, while neutral acceptance (*p* = 0.002) was a positive predictor. Fear of death and approach acceptance were not significant predictors. Collectively, these five subscales explained approximately 43% of the variance in FATCOD scores, underscoring that students who avoid thinking about death or view it primarily as an escape are less prepared for end-of-life care, whereas those who regard death neutrally as a natural part of life feel more prepared.

**Table 8.** Predictors of Caring for Dying Patients

Item	Model 1			
	Unstandardized coefficients		Standardized coefficients	
	B	SE	Beta	Sig.
Fear of death	−0.011	0.189	−0.008	0.954
Death avoidance	−1.113	0.229	−0.643	0.000^**^
Neutral acceptance	1.095	0.343	0.372	0.002^**^
Approach acceptance	−0.127	0.098	−0.118	0.197
Escape acceptance	−0.621	0.253	−0.419	0.016^*^
DAP-R total	0.096	0.125	0.245	0.445
*F* (sig)	17.447	(0.000^**^)		
*R* ^2^	0.429	42.9%		
*R*^2^ change				

^*^ Statistically significant at *p* ≤ 0.05.

^**^ Statistically significant at *p* ≤ 0.01.

## Discussion

Caring for patients with terminal illnesses is a key aspect of nursing practice. It demands both clinical expertise and emotional fortitude. Nurses must be confident and compassionate when caring for patients nearing the end of their lives. The purpose of this study is to analyze undergraduate nursing students’ views on death and their preparedness to care for dying patients.

### Nursing students’ attitudes toward death

The results of the DAP-R scale in this study indicate that nursing students generally exhibit a balanced perspective on death, with a strong disposition toward acceptance. Yet, notable levels of fear and avoidance persist. The total DAP-R score (172.00 ± 30.51) in this study suggests that the majority of students exhibit moderate to high levels of commitment with death-related attitudes. This finding suggests a complex interchange of acceptance, fear, and avoidance. When compared with similar studies conducted in different cultural contexts, the total score in this study varies significantly from those reported from Jordan (153.7 ± 21.5)^1^ and from China (89.27 ± 7.26).^15^ These variations suggest that cultural, educational, and spiritual factors may play a substantial role in influencing nursing students’ attitudes toward death and end-of-life care.

However, the comparatively high score in this study does not necessarily indicate a generally cheerful view. Instead, it emphasizes the presence of both acceptance and emotional ambivalence, which can coexist in students’ viewpoints. More in-depth examination of the DAP-R subscales reveals moderate levels of fear of death (35.84 ± 8.99) and death avoidance (26.05 ± 6.88), with a significant proportion of students scoring above 60% (68.0% and 71.3%, respectively). In a similar study in Italy, researchers addressed the importance and impact of death education for students during training. The study concluded reduced fear of death, increased acceptance, and improved attitudes toward assisting the dying.^16^

The high neutral acceptance score (29.41 ± 4.04) and Approach Acceptance score (56.24 ± 11.03) indicate that most students accept death as a natural part of life, supported by a strong belief in an afterlife. Over 83.6% of students scored ≥60% on Approach Acceptance, suggesting that spiritual and religious beliefs influence their experience of death. This is consistent with a similar study undertaken in Indonesia and Iran,^17,18^ where cultural and religious teachings promote the view of death as a transition rather than an end. In Islamic cultures, for example, death is considered as a gateway to the hereafter, which may explain why students from mostly Muslim families are more accepting and less resistant to end-of-life discussions.

Despite good views regarding death, escape acceptance (24.46 ± 8.04) is a significant component, with 62.3% of students scoring ≥60%. This implies that a large majority of students see death as a respite from pain, which may influence their attitude to palliative care. While this type of acceptance may express empathy for patients’ suffering, it also implies an emotional distance from caregiving responsibilities. This emphasizes the importance of training to strengthen coping skills and emotional resilience. The occurrence of mild escape acceptance in this study is consistent with studies from Palestine and Iran,^18,19^ where students exposed to terminally ill patients frequently show emotional pain and moral difficulties after seeing extended suffering. While escape acceptance can demonstrate empathy, it may also emphasize the need for improved emotional coping methods and resilience training in nursing curricula to ensure students retain a compassionate yet professionally balanced approach to end-of-life care.

### Nursing students’ attitudes toward caring for dying patients

The results of this study indicate that nursing students have balanced views of death itself but struggle with end-of-life care, as reflected in the FATCOD Scale score. Although previous sections noted some acceptance of death, the current FATCOD mean score (96.81 ± 11.90) is relatively low, suggesting many students still feel uncertain or negative about end-of-life care responsibilities. This finding is inconsistent with results from other studies, scoring significantly lower than other studies recruiting Jordan (93.81 ± 9.2), Indonesia (93.88 ± 5.66), Kazakhstan (94.50 ± 12.41), British (95.3 ± 9.1), Italian (101.8 ± 7.3), Spanish (108.73 ± 9.49), and Swedish (123.0 ± 10.1) nursing students.^11,17,20–22^ The score variations highlight the potential influence of cultural, educational, and health care system differences on nursing students’ attitudes toward death and dying patients.

Palliative care topic integration in nursing education is particularly weak, and clinical exposure is variable, which may contribute to worse preparation and FATCOD scores. The mixed cultural background of the current study sample and related religious context may have played a role in shaping students’ perceptions regarding death and dying. Additionally, clinical exposure in hospital settings may have influenced students’ perspectives, as hands-on interactions with terminally ill patients help them acquire emotional resilience and practical abilities in end-of-life care. A similar study in China confirmed that students exposed to graded exposure to death during clinical education improved their emotional adjustment and psychological preparedness for helping the dying patients, claiming that it also deepened their understanding of life and death.^23^

The findings also indicate that students had a more positive attitude about assisting the relatives of dying patients, underscoring the notion that comprehensive end-of-life care includes family members who are grieving and feeling loss in addition to the patient. This is consistent with previous research, which shows that nursing students realize the importance of family support during end-of-life care and advocate for compassionate measures to reduce the emotional strain on both patients and their loved ones.^22^

These findings highlight the broader concern that nursing education in the UAE provides limited emphasis on structured palliative care training. While students demonstrated a degree of acceptance of death itself, their comparatively low FATCOD scores suggest that they are underprepared to translate these attitudes into practical, compassionate care for dying patients. Similar challenges have been observed in other countries where palliative care education is underdeveloped, indicating the need for systematic curriculum reform.

### Effect of death attitudes and care for dying patients

The findings show that students’ perceptions toward death influence their approach to end-of-life care. Fear of death (*r* = −0.384, *p* < 0.001) and death avoidance (*r* = −0.506, *p* < 0.001) have significant negative correlations with FATCOD scores, indicating that students who are fearful of death or actively avoid thinking about it are less likely to feel positively about caring for dying individuals. This finding is consistent with previous research, which revealed that nursing students who had more favorable attitudes regarding death demonstrated more skill and confidence in caring for the dying and that emotional avoidance may limit students’ capacity to engage fully with the terminal patients and their families.^1,24^

Differences in gender must also be considered. The overall FATCOD scores of males and females in this study did not differ considerably, despite the fact that females scored slightly higher on death avoidance. One explanation is that although female students may be more likely to avoid thinking about death, other factors, such as clinical training, personal experiences, and cultural expectations, influence their willingness and attitudes toward providing end-of-life care, lessening the impact of death avoidance alone. This implies that significant variations in preparedness to provide compassionate palliative care are not always predicted by gender differences in DAP-R subscales.

Escape acceptance has a substantial negative connection with caring attitudes (*r* = −0.322, *p* < 0.001). This suggests that students who see death primarily as an escape may be less likely to commit emotionally or professionally in caring for persons nearing the end of their lives. In contrast, neutral acceptance (*r* = 0.130, *p* = 0.153) and approach acceptance (*r* = −0.118, *p* = 0.197) have no significant correlation with FATCOD scores, implying that a general acceptance of death, whether as a natural part of life or as a religious passage, does not directly convey into more potent attitudes toward caring for dying patients. This was also supported by previous research results.^16,18,20^

Interestingly, the overall DAP-R score correlates negatively with FATCOD (*r* = −0.337, *p* < 0.001). This suggests that unresolved or complex death attitudes, especially those rooted in fear and avoidance, may act as limitations to developing compassionate and competent caregiving skills in palliative settings. Furthermore, academic progression and exposure to clinical settings appear to influence these views, emphasizing the need to incorporate death-related education and palliative care experiences into nursing curriculum.^24,25^

### Limitations

This study has several limitations that should be considered when interpreting the findings. First, the cross-sectional approach precludes the examination of changes in attitudes over time or in response to targeted educational initiatives. Second, the use of self-reported surveys may have added social desirability bias, as students may react in ways that they believe are anticipated rather than representing their genuine feelings. Furthermore, the sample lacks diversity, limiting the findings’ generalizability to larger student populations. Last, the study did not examine personal variables such as prior experience with death, religious level, or exposure to palliative care education in depth, all of which may influence attitudes toward dying patients.

In addition, while the UAE is a predominantly Muslim country, the study did not collect data on participants’ specific religious affiliation. Instead, the discussion highlights the role of religiousness—the degree of personal belief and practice—rather than labeling participants by religion. This approach allowed us to acknowledge the cultural and spiritual context without compromising ethical standards. Moreover, despite the anonymity of the survey, students might still have answered in ways that matched anticipated professional norms, so social desirability bias cannot be totally ruled out.

## Conclusion

This study concluded that, while many students showed some acceptance of death, high degrees of fear, avoidance, and escape acceptance had a major impact on their views about end-of-life care. The study findings highlight that insufficient integration of palliative care education in the UAE contributes to students’ uncertainty and emotional strain when facing death and dying. Furthermore, the comparatively low FATCOD ratings indicate a need for better integration of palliative care instruction within nursing curricula. Structured clinical exposure, psychological coping mechanisms, and reflective learning opportunities could all be used to help students gain the essential confidence and emotional resilience.

## Recommendation

Understanding how nursing students experience death is crucial in minimizing its emotional and psychological impact. To help them maintain a positive attitude toward end-of-life care, nursing education should include focused training on death and dying. This should cover essential topics such as the physical care of dying patients, emotional support for families, ethical considerations, and students’ own beliefs and feelings about death. Hands-on learning through simulations, open discussions, and psychological support can better prepare students for these challenging experiences. In the context of the UAE, this should also involve developing formal partnerships with palliative care centers and hospices to ensure students gain direct, guided experience in end-of-life care.

## Data Availability

The datasets used and/or analyzed during the current study are available from the corresponding author upon reasonable request.

## Author Agreement

The authors have seen and approved the article, which has not been published before and is not under consideration for publication elsewhere.

## Abbreviations Used

DAP-R: Death Attitude Profile-Revised
FATCOD: Frommelt Attitudes Towards Care of the Dying
PTSD: Post-traumatic stress disorder
UAE: United Arab Emirates
